# The role of alternative polyadenylation in the antiviral innate immune response

**DOI:** 10.1038/ncomms14605

**Published:** 2017-02-24

**Authors:** Xin Jia, Shaochun Yuan, Yao Wang, Yonggui Fu, Yong Ge, Yutong Ge, Xihong Lan, Yuchao Feng, Feifei Qiu, Peiyi Li, Shangwu Chen, Anlong Xu

**Affiliations:** 1 State Key Laboratory of Biocontrol, Department of Biochemistry, School of Life Sciences, Sun Yat-Sen University, Guangzhou 510275, People's Republic of China; 2 School of Life Science, Beijing University of Chinese Medicine, Beijing 100029, People's Republic of China

## Abstract

Alternative polyadenylation (APA) is an important regulatory mechanism of gene functions in many biological processes. However, the extent of 3′ UTR variation and the function of APA during the innate antiviral immune response are unclear. Here, we show genome-wide poly(A) sites switch and average 3′ UTR length shortens gradually in response to vesicular stomatitis virus (VSV) infection in macrophages. Genes with APA and mRNA abundance change are enriched in immune-related categories such as the Toll-like receptor, RIG-I-like receptor, JAK-STAT and apoptosis-related signalling pathways. The expression of 3′ processing factors is down-regulated upon VSV infection. When the core 3′ processing factors are knocked down, viral replication is affected. Thus, our study reports the annotation of genes with APA in antiviral immunity and highlights the roles of 3′ processing factors on 3′ UTR variation upon viral infection.

## Abstract (web summary)

RNA processing by alternative polyadenylation (APA) can result in 3′UTR diversity and regulation of target RNA function. Here the authors survey APA in viral infection of macrophages.

The 3′ end of eukaryotic mRNAs mainly contains a long stretch of adenosines termed the ‘polyadenylated tail'. Post-transcriptional polyadenylation of nascent transcripts through the addition of a polyadenylated tail to the 3′-untranslated regions (UTR) is a key mRNA processing event in eukaryotic cells^1^. This reaction requires numerous trans-acting factors that are directed to poly(A) sites by *cis*-acting RNA elements within the pre-mRNA^2^,^3^. A large proportion of protein-coding genes contain more than one polyadenylation site^4^,^5^,^6^, indicating that alternative polyadenylation (APA) is a widespread biological mechanism. Alternative polyadenylation produces proteins with distinct C-terminal amino acids or mRNA isoforms with different 3′-untranslated regions (tandem 3′ UTRs) that may have an important role in regulating the function, stability, localization and translation efficiency of target RNA, as the 3′ UTR serves as the major docking platform for RNA binding proteins and microRNAs^7^. With the expansion of technologies for mapping poly(A) sites and identifying APA events, transcriptome-wide APA has been recognized as an important mechanism of gene regulation. It has become increasingly clear that poly(A) site switching is subject to dynamic regulation under diverse biological and disease conditions. The widespread shortening of 3′ UTRs by APA has been found in cancer cells^8^ as well as in proliferating cells^9^, and the lengthening of tandem 3′ UTRs has been detected during cell differentiation and in nerve cells^10^,^11^,^12^. Our earlier study also described a dynamic landscape of tandem 3′ UTRs during zebrafish development^13^ and in zebrafish spleen cells during the immune response^14^. These previous studies indicate that APA is a widespread mechanism requiring further functional investigation.

To detect virus invasion and activate antiviral/pro-inflammatory responses, eukaryotic cells express various pattern-recognition receptors (PRR). In the past two decades, virus-specific PRRs, including membrane-bound Toll-like receptors (TLR)^15^,^16^,^17^,^18^,^19^ and the cytosolic viral RNA sensing RIG-I-like receptors (RLR) have been well characterized^20^,^21^. Furthermore, potential DNA sensors, such as cyclic GMP-AMP synthase (*cGAS*)^22^, interferon-inducible protein 16 (*IFI16*)^23^ and DEAD box polypeptide 41 (*DDX41*)^24^, have been shown to monitor the cytoplasm for the presence of viral DNA. The recognition of viral nucleic acids by PRRs initiates downstream signalling cascades and leads to the production of type I interferon (IFN)^25^, which subsequently triggers interferon signalling to affect the early and late stages of the virus life cycle^26^. To maintain immune system homoeostasis, genes with antiviral functions are synchronously and temporally regulated by transcriptional ‘on' and ‘off' switches that account for the specificity of gene expression. In addition to transcriptional regulation, post-transcriptional regulation, including phosphorylation and ubiquitination, has an important part in controlling the expression levels of many antiviral immune-related genes^27^,^28^,^29^,^30^,^31^,^32^. The alternative splicing of central genes involved in antiviral pathways, such as *MYD88*, *IRAK1*, *IKKɛ* and *IRF3*, has also been demonstrated to negatively regulate antiviral responses^33^,^34^,^35^,^36^.

Although APA is involved in numerous biological processes, its role in antiviral immunity is unexplored. The goal of this study is to explore the global APA profile and to characterize the dynamic APA-mediated regulation of antiviral responses using *in vitro* transcription-sequencing APA sites (IVT-SAPAS), a much improved approach of SAPAS^37^. The global APA profile in response to viral infection may provide insight into the function of APA in the antiviral innate immune response and present a genomic view of the APA-mediated regulation of gene expression during this response.

## Results

### Transcriptome-wide analysis of the 3′ end of mRNA

To determine whether APA is implicated in the antiviral immune response, we infected human monocyte-derived macrophages (MDM) and mouse peritoneal macrophages with vesicular stomatitis virus (VSV) (Supplementary Fig. 1). The MDMs were obtained from an apparently healthy donor using magnetic CD14 microbeads followed by stimulating with rhM-CSF for 7 days. Peritoneal macrophages were obtained from C57BL/6J mice. Both types of macrophages were infected with VSV at 0.5 multiplicity of infection (MOI), and cells were collected at 0, 2, 4, 8, 16 and 24 h. The transcription and secretion of *IFN-beta* and *RANTES* were measured using qRT-PCR and ELISA, respectively, to evaluate the stimulation of antiviral responses. The results showed that the mRNA abundance and protein secretion of *IFN-beta* and *RANTES* increase significantly upon VSV infection, indicating that the cells were efficiently infected and adequate for IVT-SAPAS library construction (Supplementary Fig. 2).

**Figure 1:**
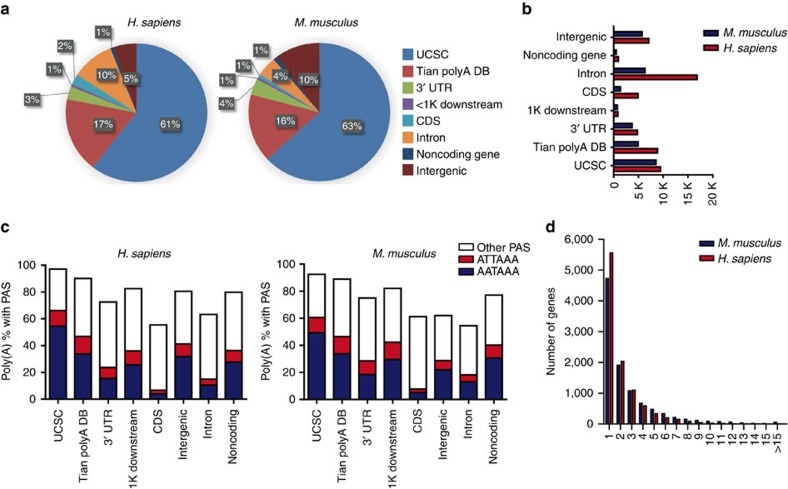
Characteristics of IVT-SAPAS data. (**a**) Genomic locations of reads uniquely mapped to the nuclear genome after internal priming filtering. (**b**) Genomic locations of the poly(A) sites. Only poly(A) sites with at least five normalized reads were considered. (**c**) Poly(A) signal hexamer (PAS) distributions for poly(A) sites from different genomic locations. Canonical PAS were rarely found for poly(A) sites located in CDS. (**d**) Number of genes with different numbers of tandem poly(A) sites.

After high-throughput sequencing, 257,957,488 and 564,259,676 raw reads were obtained from the human and mouse samples, respectively. By mapping and filtering internal priming at A-rich internal regions as previously described^13^,^37^, 90,281,481 and 181,398,638 reads were obtained and used for downstream analysis (Supplementary Tables 1 and 2). More than 80% of the qualified reads were mapped to annotated 3′ UTRs or 1-kb downstream regions (Fig. 1a). The pooled data across all time points were used to identify 53,697 poly(A) sites in human and 31,820 in mouse, in which each poly(A) site had five or more normalized reads, as the total read counts from each time point were normalized to 1 million. More than 34.02% of the poly(A) sites in human and 42.45% of the sites in mouse were mapped to known poly(A) sites in the UCSC transcript ends database and Tian's database (Fig. 1b). Two canonical hexanucleotide polyadenylation signal sequences, AAUAAA or AUUAAA^38^,^39^, which were mostly identified 10–30 bases upstream of the cleavage/polyadenylation site, were less used in coding sequences (CDSs) (Fig. 1c). This was similar to the results of a previous study^13^, which suggested that there may be a novel polyadenylation mechanism in coding regions. In addition, 10043 and 10179 UCSC canonical genes with at least one normalized poly(A) site in the final exon were identified in human and mouse samples, respectively. In accordance with Tian's study, which showed that 54% of human genes and 32% of mouse genes possess multiple alternative poly(A) sites^40^, we found that 52.91% of the identified human genes and 45.4% of the mouse genes contained more than one poly(A) site (Fig. 1d).

### 3′ UTR variation in human and mouse upon viral infection

**Figure 2:**
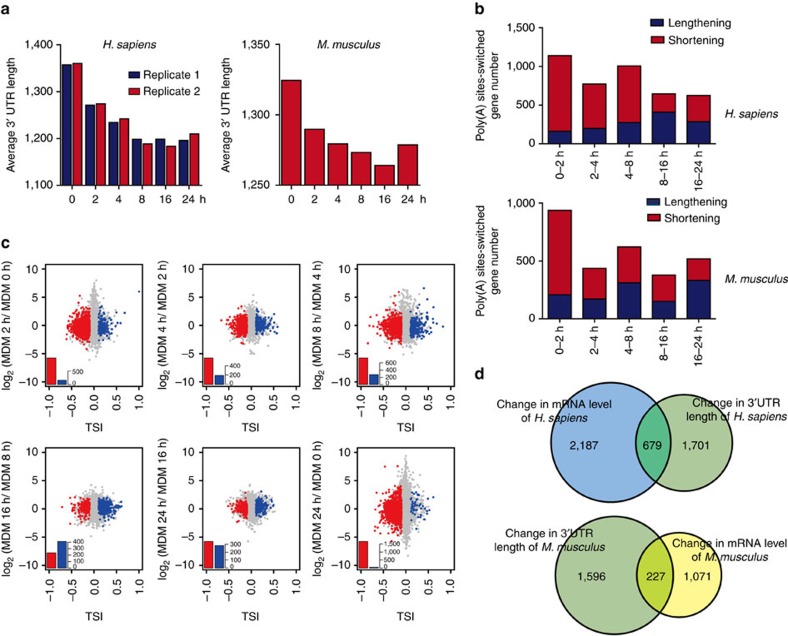
Characteristics of tandem 3′ UTRs during the anti-VSV immune response. (**a**) Characteristics of the average tandem 3′ UTR length during the anti-VSV immune response. Human and mouse showed similar patterns. (**b**) The number of poly(A) site-switched genes at consecutive time points in human MDMs upon VSV infection. (**c**) Poly(A) site switching at consecutive time points in human MDMs upon VSV infection. The *x* axis denotes TSI, and a larger positive value indicates that longer tandem 3′ UTRs were observed at later time points. Genes with significant switching to longer (blue) or shorter (red) tandem 3′ UTRs at later time points (FDR=0.01) are coloured accordingly. The *y* axis denotes the logarithm of the expression level of genes from later time points relative to earlier samples. (**d**) Venn diagram showing the overlap of DEGs and poly(A) site-switched genes.

Since IFN-beta secretion by infected cells is a critical determinant of cellular susceptibility to viral infection, we analysed the mRNA abundance of *IFN-beta* in our data and found peak transcription to be at 4 h post infection, suggesting a remarkable antiviral response (Supplementary Table 3). We then defined the 3′ UTR length of a gene at a single time point as the average length of tandem 3′ UTRs weighted by the 3′ UTR isoform expression levels and found that the average 3′ UTR length shortened after viral infection in both species (Fig. 2a). Biological replicates of VSV-infected MDMs showed a high correlation (Supplementary Fig. 3) and presented a consistent pattern (Fig. 2a).

By counting the number of APA genes between two consecutive time points, we showed that the number of APA genes was highest from 0 to 2 h, but declined slightly then increased from 4 to 8 h (Fig. 2b). We further analysed the 3′ UTR variation of genes possessing more than one poly(A) site in their 3′ UTRs between two consecutive time points (Fig. 2c; Supplementary Fig. 4). The number of shortened genes increased from 0 to 8 h in human, while the number of lengthened genes increased at later time points (false discovery rate (FDR)=0.01 and tandem 3′ UTR isoform switch index (|TSI|)>0.1). A similar profile was observed in mouse, with the number of shortened genes increasing from 0 to 4 h post infection, while the number of lengthened genes increased at later time points. In all, human and mouse showed a similar pattern of tandem 3′ UTR dynamics during the antiviral immune response, and the similar profiles suggested that APA may be a general regulatory strategy for antiviral responses in mammals.

### Genes of APA or differential expression upon viral infection

In addition to measuring tandem 3′ UTR length, our data allowed us to determine mRNA transcription levels by totalling the reads that fell into the 3′ UTR of a given gene. We identified 2,866 human genes and 1,298 mouse genes with mRNA abundance changes greater than three-fold (FDR-adjusted *P*<0.01, Fisher's exact test). However, the genes with altered mRNA abundance showed little overlap with the genes displaying APA switching. Only 23.7% of the human and 17.5% of the mouse genes that showed changes in mRNA abundance simultaneously switched poly(A) sites during the antiviral immune response (Fig. 2d), suggesting no obvious correlation between the changes in mRNA abundance and APA.

To identify the function of the genes that switched poly(A) sites during the antiviral immune response, we performed gene ontology (GO) analysis of human APA genes between two consecutive time points on the DAVID website using the genes expressed at all time points as the background^41^. Interestingly, we found the genes that changed their 3′ UTR length in the first 2 h had little association with antiviral immune response. However, immune-related GO categories, such as response to dsRNA, inflammatory response, defence response and response to virus, were significantly enriched from 2 h post VSV infection, especially from 4 to 8 h and 8 to 16 h. APA genes were also enriched in protein transport, protein localization and categories associated with adaptive immunity, such as T-cell proliferation, from 8 to 24 h after viral infection. Interestingly, in the later period of viral infection, genes related to apoptosis significantly switched their poly(A) sites (Table 1).

To investigate whether the differentially expressed genes (DEGs) also functioned in immune-related biological processes, we performed GO analysis and found that the genes with altered mRNA levels were enriched in defence response, inflammatory response, immune response, response to virus and other immune-related biological processes both in human and mouse (Table 2). Collectively, the organisms systematically regulated a variety of biological processes to combat virus invasion, and GO enrichment analysis revealed that APA played an important role in the entire antiviral immune response.

### APA is involved in virus recognition and eradication

**Figure 3:**
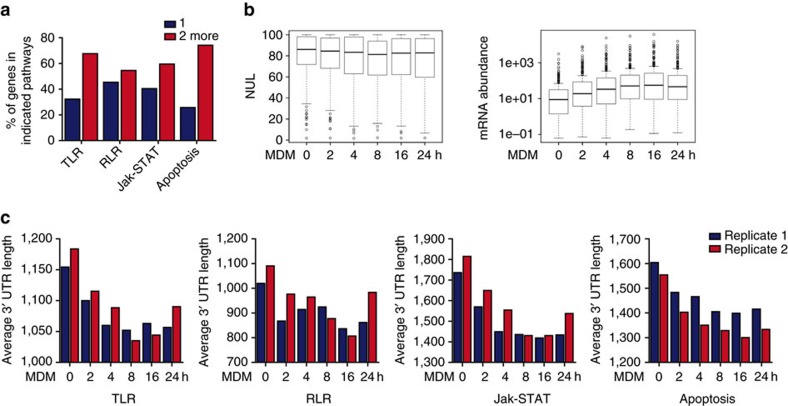
APA is involved in the innate antiviral immune response. (**a**) Percentage of genes with two or more tandem 3′ UTRs in the indicated pathways. (**b**) Boxplot of normalized weighted 3′ UTR (NUL) (upper panel) and the mRNA abundance of ISG (lower panel). The distribution is shown using box plots. Horizontal line indicates median and error bars indicate range. (**c**) Average 3′ UTR length of genes involved in the indicated pathways during the antiviral immune response as determined from two biological replicates of IVT-SAPAS.

Viral infection is detected by germline-encoded PRR, which initiate innate antiviral immune responses, including the activation of IFNs and pro-inflammatory cytokines. The recognition of viral RNA activates at least three main downstream signalling pathways, the TLR signalling pathway, the RLR signalling pathway and the Jak-STAT signalling pathway^42^. Moreover, apoptosis, which genetically controls cell death during the development and homoeostasis of multicellular organisms, can also remove virus-infected cells to suppress viral replication and therefore plays an important role in the maintenance of an effective immune system^43^,^44^,^45^,^46^,^47^. Here, we revealed that, in human, 67.7% of the genes involved in the TLR signalling pathway, 54.5% of the genes involved in the RLR signalling pathway, 59.6% of the genes in the Jak-STAT signalling pathway and 74.2% of the genes involved in apoptosis possessed more than one tandem 3′ UTR (Fig. 3a), suggesting that these pathways might be regulated through APA in some respects. Further analysis of the TLR, RLR and Jak-STAT pathways revealed that most of these genes switched poly(A) sites to produce 3′ UTR variation following viral infection (Supplementary Fig. 5). However, *IFNB1*, *TICAM1*, *STAT1*, *IRF7*, *IKBKB*, *MAVS*, *TYK2*, *PIAS4*, *IL15RA* and *IFNGR1*, which play pivotal roles in antiviral immune signalling, have a single poly(A) site located in their 3′ UTRs and exhibited altered mRNA abundance upon viral infection (Supplementary Tables 3 and 4), suggesting that the *de novo* transcription regulation of genes without APA may have a greater impact on the participation of these genes in the antiviral response. Given that the genes in immune-related pathways possess APA, we extended our analysis to interferon-stimulated genes (ISG) selected from previous studies^48^,^49^ and focused on the average normalized 3′ UTR length of hundreds of ISGs. The results showed that a high proportion of ISGs express longer 3′ UTRs in resting cells but express shorter 3′ UTRs following viral invasion (Fig. 3b, left panel). The mRNA abundance levels of the ISGs increased, and the average expression level peaked at 16 h post infection (Fig. 3b, right panel). Interestingly, we observed that the average 3′ UTR length of the genes involved in the above pathways shortened upon VSV infection, similar to the pattern illustrated in Fig. 2a (Fig. 3c).

**Figure 4:**
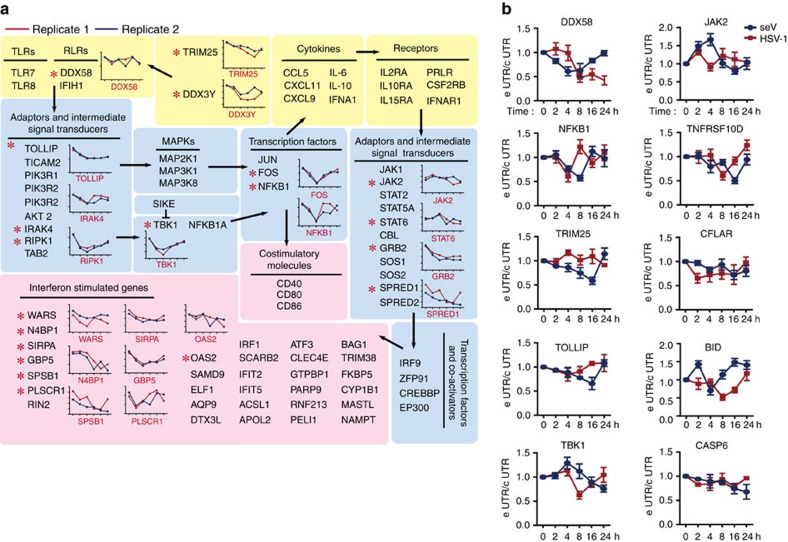
Genes with switched poly(A) sites involved in antiviral signalling network. (**a**) The genes with APA involved in virus recognition and signal transduction were shown. The black arrows indicate signal transduction. The line graphs illustrate duplicate data, the *x* axis denotes six infection time points and the *y* axis denotes normalized 3′ UTR length. The yellow background indicates the cytokine and receptor layer, the blue background indicates the adaptor and intermediate signal transducer layer and the pink background indicates the terminal effectors. * Indicates the APA genes shown in the line graph. (**b**) Real-time PCR analysis of the selected genes with APA upon SeV or HSV-1 infection in THP-1 cells. The *x* axis denotes six time points post infection and the *y* axis denotes e UTR/c UTR, which represents the ratio of longer 3′ UTR isoforms to total mRNA isoforms. Data are presented as mean±s.d. of three independent experiments.

To add evaluation data, we validated the APA of 20 selected immune-related genes in two biological replicates and, as shown in Fig. 4a, these data correlated well. Moreover, such APA events were also confirmed by qRT-PCR assays (Supplementary Fig. 6). To evaluate whether APA is a general phenomenon in cells after viral infection, and considering that the genes involved in the above-mentioned pathways switched poly(A) sites upon VSV infection, THP-1 cells were infected with another RNA virus, Sendai virus (SeV) or a DNA virus, type I herpes simplex virus (HSV-1), and then qRT-PCR assays were performed to test the 3′ UTR usages of 10 selected genes. The results showed that all the selected genes switched poly(A) sites (Fig. 4b), suggesting that APA is a general phenomenon that occurs after viral infection in macrophages. Taken together, APA participates in the antiviral innate immune response and might influence signal transduction.

### The impact of tandem 3′ UTRs on protein production

**Figure 5:**
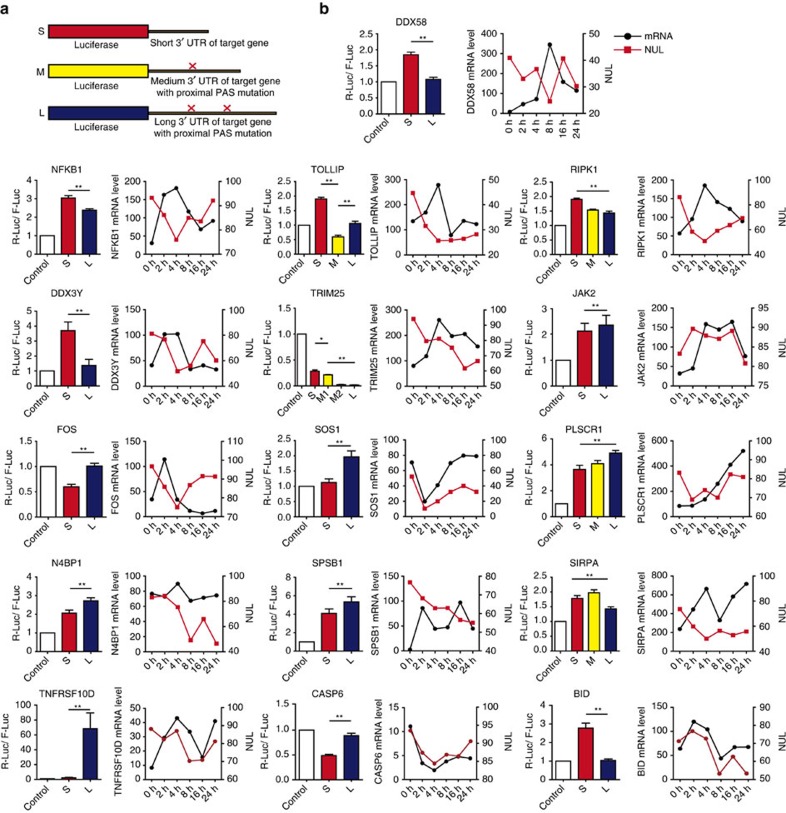
Functional consequences of expressing short or long mRNA isoforms. (**a**) Schematic diagram of psiCHECK-3′ UTR constructs. S=short 3′ UTR; M=medium 3′ UTR with proximal poly(A) signal mutation; L=long 3′ UTR with proximal and middle poly(A) signal mutations. (**b**) psiCHECK-2 or psiCHECK-3′ UTR constructs were transfected into 293T cells. The luciferase expression of a reporter possessing the 3′ UTRs of target genes was normalized to that of psiCHECK-2. Histograms show the mean reporter activity values, with error bars indicating s.d. (*n*=9, performed as three independent experiments with three transfections of each reporter, *P<0.05 and **P<0.01 as determined by the Newman–Keuls *post-hoc* test). The line graphs show the mRNA abundance level or normalized change in 3′ UTR length (NUL) of the target genes from the sequencing data. The *x* axis denotes six infection time points and the left *y* axis denotes mRNA abundance levels, while the right *y* axis denotes normalized 3′ UTR length.

To evaluate whether the variation in 3′ UTR usage affects protein production, thirteen genes that exhibited poly(A) site switching during the antiviral immune response, *TOLLIP*, *FOS*, *NFKB1*, *DDX58*, *RIPK1*, *DDX3Y*, *TRIM25*, *JAK2*, *SOS1*, *N4BP1*, *SIRPA*, *SPSB1* and *PLSCR1*, were selected for further analysis. Because the poly(A) signal plays a pivotal role in polyadenylation, a series of constructs were made to test how distinct 3′ UTRs affect the protein output of target genes in a luciferase activity assay. The shortest 3′ UTR with the proximal poly(A) site of the target genes was inserted into the psiCHECK-2 plasmid downstream of the *Renilla* luciferase translational stop codon and named the S construct. Similarly, construct M indicated a middle length 3′ UTR with both proximal and middle poly(A) sites, but the proximal poly(A) site was mutated to avoid the formation of the S isoform. Meanwhile, construct L contained all the putative poly(A) sites, but the proximal and middle poly(A) sites were both mutated to avoid the formation of the S and M isoforms. (Fig. 5a). Luciferase reporter assays indicated that tandem 3′ UTRs indeed affected luciferase activity (Fig. 5b). Previous studies have revealed *DDX58* (also known as *RIG-I*) to be a cytoplasmic viral RNA detector and crucial to the eradication of replicating viral genomes^50^,^51^,^52^. Our results here further showed that use of the short 3′ UTR of *DDX58* enhanced *Renilla* luciferase protein expression (Fig. 5b). As the endpoint of a series of signal transduction events initiated by a vast array of stimuli, *NFκB1* switched its poly(A) site during the antiviral immune response, and its short 3′ UTR also enhanced *Renilla* luciferase protein production (Fig. 5b). Similar patterns were observed for *TOLLIP*, *RIPK1*, *DDX3Y* and *TRIM25*, the protein expression levels of which increased when the short 3′ UTRs were used. However, with the exception of *SIRPA,* the other selected genes showed an opposite pattern (Fig. 5b). To confirm the estimated outputs from 293T cells, two other cell lines, A549 cells and Hela cells, which are widely used in the study of immunity and APA^53^,^54^, respectively, were chosen for parallel luciferase assays. The results showed that the effects of the distinct 3′ UTR isoforms on protein outputs were consistent among these cell lines (Supplementary Fig. 7).

Owing to the distinct miRNA and RBPs, which can bind to target mRNAs, tandem 3′ UTRs may influence protein outputs not only by affecting mRNA stability but also by affecting translational efficiency. To explore these possibilities, we transfected reporter gene constructs into 293T cells and performed qRT-PCR. The results showed that nine of the selected genes (*DDX58*, *NFκB1*, *TOLLIP*, *RIPK1*, *DDX3Y*, *SOS1*, *N4BP1*, *SIRPA* and *BID*) had mRNA expression patterns similar to the protein expression patterns, while the rest of the genes exhibited discrepant patterns (Supplementary Fig. 8). Thus, our results showed that tandem 3′ UTRs could influence mRNA abundance, which further affected protein production. To determine whether tandem 3′ UTRs can also affect translation efficiency, we performed a polysome profiling assay, which is a widely used and well-established experimental procedure, to estimate the translation efficiency of the endogenous mRNAs for six pivotal genes (*DDX58*, *NFκB1*, *TRIM25*, *TOLLIP*, *DDX3Y* and *BID*). Total RNA was isolated from each fraction of the polysome profile, then a common primer pair that detected both isoforms generated by proximal and distal poly(A) site usage and extended primer pair that only detected the isoform generated by cleavage at the distal poly(A) site was used to perform qRT-PCR. The specified mRNA level in each fraction was calculated as a percentage of the total. Our results showed that the levels of the tandem 3′ UTR isoforms of the *DDX58* and *NFκB1* mRNAs did not differ significantly among fractions, suggesting similar translation efficiencies. In contrast, for the remaining genes, the polysome-bound fraction consisted of a higher proportion of the short 3′ UTR isoforms than the long 3′ UTR isoforms (Supplementary Fig. 9), suggesting higher translation efficiency of short 3′ UTR isoforms. Collectively, these results suggested that tandem 3′ UTRs could affect protein expression both by influencing mRNA abundance and translation efficiency.

### APA increases biological complexity of antiviral immunity

To determine if mRNA abundance change and tandem 3′ UTR variation act synergistically to increase biological complexity by promoting the multi-functionality of existing proteins, we next analysed the sequencing data and the reporter assay results. As for *DDX58*, *NFκB1*, *TOLLIP*, *RIPK1*, *DDX3Y*, *TRIM25* and *JAK2*, which are involved in antiviral signalling transduction, both increased mRNA abundance and switched poly(A) sites eventually led to enhanced protein production following viral infection (Fig. 5b). Thus, we assumed that tandem 3′ UTR variation and mRNA abundance change are both involved in increasing the protein expression levels of genes that serve as signal transduction components to effectively regulate antiviral responses. In contrast, some ISGs, including *PLSCR1*, *N4BP1*, *SIRPA* and *SPSB1*, exhibited increased mRNA levels but switched poly(A) sites, resulting in lower protein production (Fig. 5b). Thus, we assumed that for genes that may serve as antiviral effectors, APA may be a complicated mechanism to moderate protein expression to avoid an over immune response. In all, we suggest that variation in tandem 3′ UTRs functions synergistically with changes in mRNA abundance to increase the biological complexity of antiviral responses.

### APA influences viral replication

Recently, underlying regulatory mechanisms of APA have been raised, including the following: (1) The strength of *cis*-elements, (2) The concentration and activity of 3′ processing factors and (3) The concentration and activity of regulatory factors. The 3′ processing complex in mammalian cells is consists of over 20 core proteins^55^, including cleavage and polyadenylation specificity factor (CPSF), cleavage factor I (CFIm) and cleavage factor II (CFIIm). In addition, *SYMPK*, poly(A) polymerase (*PAP*), poly(A) binding protein nuclear 1 (*PABPN1*), two cytoplasmic poly(A) binding protein (*PABPC1* and *PABPC4*), and RNA polymerase II C-terminal domain (*RNAPII*) are also involved in this process^56^,^57^.

**Figure 6:**
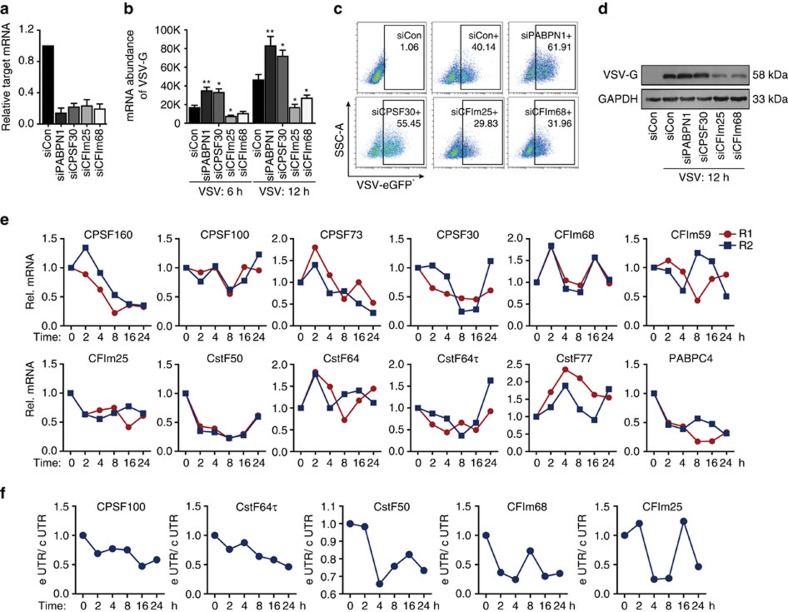
APA influences viral infection. (**a**) siRNAs were transfected into A549 cells, and the interference efficiency was determined with qRT-PCR at 48 h. (**b**) We transfected A549 cells with individual siRNAs for 48 h and infected them with VSV-eGFP at an MOI of 1 for 6 h or 12 h. Then, qRT-PCR was performed to detect VSV replication at the mRNA level. Data are mean±s.d. *P<0.05; **P<0.01. (**c**) FACS analysis of A549 cells transfected with control siRNA (siCon) or the indicated siRNAs and then infected with VSV-eGFP at an MOI of 2 for 6 h. FACS gating strategies from one representative experiment have been provided as Supplementary Fig. 10. (**d**) A549 cells were transfected with individual siRNAs for 48 h and infected with VSV at an MOI of 1 for 12 h. Then, western blotting was performed to detect VSV replication at the protein level. The uncropped scans of western blots have been provided as Supplementary Fig. 11. (**e**) Relative mRNA expression of the core 3′ processing factors in VSV-infected MDMs. Data were obtained from two biological duplicates of IVT-SAPAS. The *x* axis denotes six time points post infection and the *y* axis denotes relative expression. (**f**) Relative 3′ UTR length of the core 3′ processing genes with switched poly(A) sites during the antiviral immune response. Data were obtained from IVT-SAPAS. The *x* axis denotes six infection time points and the *y* axis denotes e UTR/c UTR, which represents the ratio of longer 3′ UTR isoforms to total mRNA isoforms.

Several studies have noted that knockdown of some 3′ processing factors could lead to significant changes in poly(A) site choice. For example, a study from Jenal *et al*.^58^ showed that loss of *PABPN1* resulted in extensive 3′ UTR shortening. A similar pattern was also observed in studies investigating *CFIm68* and *CFIm25* (ref. 53, 59). Conversely, Yao's^54^ study revealed that depletion of the *CstF64* paralog *CstF64τ* leads to great changes in APA, most of which were characterized by the increased relative use of distal poly(A) sites. Moreover, Thomas *et al*. reported that poly(A) site choice in a large majority of Arabidopsis genes was altered in a *CPSF30* mutant^60^. Thus, to gain some mechanistic insight into how viral infection causes APA, we firstly knocked down four key 3′ processing factors (*PABPN1*, *CPSF30*, *CFIm25* and *CFIm68*) to test whether viral proliferation was affected. After transfection with efficient siRNA, A549 cells were infected with GFP-tagged VSV at an MOI of 1 for 6 h or 12 h. Then, qRT-PCR, western blotting and flow cytometry analyses were performed to show that when the expression of *PABPN1* and *CPSF30* was disturbed, enhanced VSV replication was detected. However, *CFIm25* and *CFIm68* knock down caused the opposite result (Fig. 6a–d; Supplementary Figs 10 and 11). These data suggest that when genome-wide poly(A) site choice is perturbed, the cellular resistance to viral infection will change, indicating that APA plays a critical role in the antiviral response.

To further investigate the relationship between 3′ processing factors and the antiviral response, we then analysed changes in the expression of 3′ processing factors in our IVT-SAPAS data. As shown in Fig. 6e, we found that the expression of most 3′ processing factors was down-regulated upon VSV infection, especially *CPSF160*, *CPSF73*, *CstF50* and *PABPC4*. Thus, changes in the expression of 3′ processing factors may be one of the reasons underlying genome-wide APA shortening upon viral infection. To test whether changes in the expression of 3′ processing factors were controlled by the IFN-related pathway, THP-1 cells were incubated with *IFNα* or *IFNβ*, and qRT-PCR assays were performed. The results showed no significant changes in most 3′ processing factors upon *IFNα* or *IFNβ* stimulation (Supplementary Fig. 12), indicating that the expression of 3′ processing factors is IFN independent. Further analyses by knocking down key adaptors (*TRIF*, *MyD88* and *MAVS*) that play pivotal roles in the TLR and RLR pathways also showed no significant changes in the expression of 3′ processing factors post VSV infection (Supplementary Fig. 13). Moreover, no binding sites for immune-related transcriptional factors, such as *NFκB*, the IRFs or the STATs, were identified in the upstream regions of these 3′ processing factors. Interestingly, we found that five core 3′ processing factors (*CPSF100*, *CstF64τ*, *CstF50*, *CFIm68* and *CFIm25*) switched poly(A) sites upon viral infection (Fig. 6f), and these genes may escape from miRNA binding when short 3′ UTRs were used. Thus, we concluded here that the low mRNA abundance of 3′ processing factors may be one of the reasons underlying genome-wide APA when cells are infected with viruses. However, such mRNA changes may not be controlled by the innate antiviral pathway.

## Discussion

To understand antiviral defence, previous studies have investigated various aspects of systems virology. Microarrays were firstly used to evaluate the mRNA abundance change that occurred in a CD4^+^ T-cell line infected with HIV^61^. Subsequently, genomic analysis of the increased host immune and cell death responses induced by the 1918 influenza virus was completed^62^. In addition, a genome-wide gene expression profile was performed in the mouse BV2 microglial cell line using microarray analysis to better understand microglia–RABV interactions at the transcriptional level^63^. Together, these studies focused on the transcriptional landscape of the host response to infection. In recent years, advances in technology allowing the profiling of APA sites have provided researchers with the opportunity to identify tandem 3′ UTR variations in yeast, *Drosophila*, zebrafish, mouse and human^40^,^64^,^65^,^66^. Since APA is recognized as an important layer of gene regulation, we for the first time investigated the genome-wide APA sites in the antiviral immune response with our recently developed IVT-SAPAS method, which enriched our understanding of the host response to viral infection and the complexity of gene regulation. In this study, we presented a global picture of the tandem 3′ UTR pattern during the antiviral response, which was characterized by gradual 3′ UTR shortening. Moreover, it was interesting to find that the number of APA genes in human and mouse showed similar characteristics, such as two peaks from 0 to 2 h and 4 to 8 h, suggesting that APA is regulated in an orderly fashion and that the regulatory mechanism may be conserved across species. Our observation is consistent with Gruber's^67^ study, which showed that the 3′ UTR shortening process in dividing cells was conserved between mouse and human.

However, no clear correlation was observed between tandem 3′ UTR and mRNA abundance on a genome-wide scale. By performing GO analysis of APA genes and DEGs, we found that APA systematically regulated a variety of biological processes to participate in the antiviral immune response. Meanwhile, the DEGs were significantly enriched in immune-related categories, indicating that the organism not only directly altered mRNA abundance but also switched poly(A) sites to combat virus invasion. Further analysis of antiviral innate immune-related pathways demonstrated that APA regulates genes involved in the antiviral immune response to influence cellular resistance to viral infection. Furthermore, we showed that tandem 3′ UTRs can influence protein production both by affecting mRNA abundance and translational efficiency. Though Gruber's^67^ study revealed that global 3′ UTR shortening had a limited effect on protein abundance in proliferating T cells, our study here showed that the impact of tandem 3′ UTRs on protein production may depend on the gene. Recently, Berkovits *et al*.^68^ also reported that tandem 3′ UTRs act as scaffolds to regulate membrane protein localization, expanding our knowledge of the effects of tandem 3′ UTRs. Whether APA of the genes investigated in this study confers different subcellular localizations and functions needs further investigation.

Recently, an increasing number of studies have reported changes in genome-wide APA in many biological and physiological processes, including our research here. However, the function of APA in a specific biological process has rarely been investigated Masamha *et al*. identified *CFIm25* as a broad repressor of proximal poly(A) site usage. When *CFIm25* was depleted, cell proliferation was increased^53^. Here, we showed that when four key 3′ processing factors that alter genome-wide poly(A) site choice were knocked down, VSV replication was clearly influenced. Although the down-regulation of many 3′ processing factors was observed when cells were infected by VSV, our results showed that such changes in mRNA abundance may not be controlled by antiviral immune-related pathways. Because mammalian 3′ processing factors may undergo extensive post-translational modification, such as arginine methylation, lysine sumoylation, lysine acetylation, and serine, threonine and tyrosine phosphorylation^69^, further analysis of whether the antiviral-related pathways can regulate the post-translational modification of 3′ processing factors needs further investigation.

In all, our study here not only provides a global picture of tandem 3′ UTR patterns during the antiviral immune response but also suggests that APA may coordinate with mRNA abundance change to increase the biological complexity of antiviral responses. Moreover, our study here highlighted the roles of 3′ processing factors in the antiviral response. Further functional analysis of genes with different APA isoforms *in vivo* will expand our knowledge of innate antiviral immunity at the post-transcriptional level. Moreover, further analysis of how the expression of 3′ processing factors is regulated when cells are infected with virus will broaden our understanding of the contribution of APA on gene regulation during a specific biological process.

## Methods

### Isolation of MDMs

Human peripheral blood mononuclear cells (PBMCs) were isolated from blood of anonymous donors (Zhongshan School of Medicine, Sun Yat-sen University) by density gradient centrifugation using Lymphoprep (AXIS-SHIELD). Individuals with concurrent autoimmune disease, hepatitis virus, HIV-1 or syphilis were excluded. All samples were anonymously coded in accordance with local ethical guidelines (as stipulated by the Declaration of Helsinki), and written informed consent was obtained and the protocol was approved by the Review Board of Zhongshan school of Medicine, Sun Yat-sen University. Monocytes were separated by positive selection using magnetic CD14 microbeads according to the manufacturer's protocol (Miltenyi Biotech). Cells were collected, washed and resuspended in RPMI 1640 supplemented with penicillin, streptomycin, 10% heat-inactivated fetal bovine serum and 50 ng ml^−1^ rhM-CSF (R&D Systems). Monocytes were differentiated into MDMs for 7 days, with fresh culture medium being added every 2 days.

### Isolation of mouse peritoneal macrophages

Eight six-week-old female C57BL/6J mice were killed by cervical dislocation and sterilized by 70% ethanol, then unstimulated peritoneal macrophages were collected by washing the peritoneal cavity with washing buffer (1 × phosphate-buffered saline (PBS) wash solution containing 0.1% bovine serum albumin and 0.5 mM ethylenediaminetetraacetic acid) for eight times with 1 ml washing buffer every time. The extracted solution was centrifuged at 300 *g* for 10 min and isolated cells were washed twice with PBS and resuspended in complete medium (DMEM plus 10% heat-inactivated fetal bovine serum, 100 U ml^−1^ penicillin and 100 μg ml^−1^ streptomycin). The cells were seeded at a density of 5 × 10^5^ cells ml^−1^ in 25 cm^2^ flasks (6 ml per flask) and incubated at 37 °C in a humidified atmosphere of 5% CO_2._ After 2 h incubation, unattached cells were discarded and attached macrophages were further cultured in fresh complete medium. The Institutional Animal Care and Use Committee of Sun Yat-sen University, PRC, approved all the experimental protocols concerning the handling of mice.

### Viral infection

MDMs and mouse peritoneal macrophages were infected with VSV (strain Indiana, ATCC) at 0.5 MOI in serum-free DMEM for 1 h, washed by 1 × PBS, and incubated with fresh complete medium for various periods of time (0, 2, 4, 8, 16 and 24 h). THP-1 cells were infected with SeV (1MOI) or HSV-1 (5MOI) for the indicated hours. In this study, cells without viral infection were collected as the 0 h sample.

### IVT-SAPAS library preparation

The IVT-SAPAS libraries were constructed based on previously developed SAPAS methods^70^. Briefly, 200 ng RNA was randomly fragmented by heating, and the first round of reverse transcription was performed using an anchored oligo d (T) tagged with an Illumina A adaptor and a T7 promoter. The second strand was synthesized with RNase H, *Escherichia coli* DNA polymerase and DNA ligase. Subsequently, the RiboMAX Large Scale RNA Production System-T7 (Promega) was used to perform *in vitro* transcription according to manufacturer's instructions (Promega). The RNA product was purified with the Agencourt RNAClean XP kit (Beckman Coulter). A second round of reverse transcription was conducted using random primers tagged with part of the Illumina B adaptor. PCR was then performed to amplify the cDNA. Finally, 200–500 bp fragments were purified with AMPure XP Beads (Beckman Coulter), and the quality of the library was evaluated with an Agilent 2100 Bioanalyzer.

### Plasmid construction and luciferase reporter gene assay

The 3′ UTRs of human target genes were amplified from the THP-1 genome by a proofreading Pfu polymerase (Takara) and sequenced to confirm authenticity. The mutation of proximal poly(A) signals was performed using a Site-directed Gene Mutagenesis Kit (ExCell Bio), and 3′ UTRs of differing lengths were cloned into psiCHECK-2 plasmids. For reporter gene assays, 293T cells were plated at a density of 10^5^ cells per well in a 48-well plate. The cells in each well were transiently transfected with psiCHECK-2 plasmids with different 3′ UTRs downstream of the *Renilla* luciferase gene using ViaFect Transfection Reagent (Promega) for 24 h. Subsequently, cells were lysed and collected for luciferase reporter gene assays. The luciferase activity of cell lysates was measured with a dual luciferase reporter gene assay system (Promega) according to the manufacturer's instructions. Firefly luciferase reporters co-existing on psiCHECK-2 plasmids were used as an internal control. Each experiment was performed in triplicate and repeated three times. Data are presented as the mean±s.d.

### Bioinformatics analysis

Raw reads were first trimmed, filtered and mapped to the human or mouse genome (hg19 and mm9 downloaded from UCSC genome bioinformatics). The unique mapped reads were used for internal priming filtering by examining the genomic sequence 1 to 20 bases downstream of the poly(A) cleavage sites. To eliminate bias introduced by unequal read counts at different time points, the total counts from each time point were normalized to one million. Poly(A) site definition and tandem UTR annotation were performed as previously described^13^. Isoform-weighted 3′ UTR length was defined as the mean 3′ UTR length weighted by read counts from each tandem 3′ UTR isoform, and normalized 3′ UTR length was defined as the percentage of the isoform-weighted 3′ UTR length relative to the longest tandem 3′ UTR length across all time points. We used a linear model to assess APA switching events, and a gene with an absolute value of tandem 3′ UTR switching index (TSI) >0.1 and a *P*-value smaller than the threshold corresponding to a BH-sense FDR of 0.01 was defined as an APA gene.

### RNA interference

LipoRNAiMAX (Invitrogen) was used for transfection of siRNAs (30 nM) into A549 cells according to the manufacturer's instructions. The sequences of siRNAs are as follows:

*PABPN1*-siRNA: 5′-GUAGCUCUGGACUGAGGAA-3′; *CPSF30*-siRNA: 5′-GUGCCUAUAUCUGUGAUUU-3′; *CFIm25*-siRNA: 5′-CCUCUUACCAAUUAUACUU-3′; *CFIm68*-siRNA: 5′-GGAUCAAGACGUGAACGAU-3′.

### Immunoblot analysis

Cells were lysed with cell lysis buffer (Cell Signaling Technology) supplemented with protease inhibitor ‘cocktail' (Roche). Protein concentrations in the extracts were measured by BCA assay (Pierce). Overall, 40 μg proteins were separated by sodium dodecylsulfate–polyacrylamide gel electrophoresis (SDS–PAGE) followed by electrotransfer to polyvinylidene difluoride membrane (Hybond-P; GE Healthcare Life Sciences). Membranes were probed using indicated antibodies against VSV-G (Abcam, 1:1,000), GAPDH (Proteintech, 1:5,000), followed by the HRP-conjugated second antibody (Cell Signaling Technology, 1:10,000). Bands were revealed with Immobilon ECL kit (Millipore) and recorded on X-ray films (Kodak, Xiamen, China).

### Statistical analysis

Unless indicated otherwise, all data were presented as mean±s.d. Statistical analysis was performed using GraphPad Prism 4.0 (GraphPad Software, San Diego, CA, USA). One-way ANOVA, followed by the Newman–Keuls *post-hoc* test was used to compare groups, with *P*<0.05 considered significant.

### Data availability

Sequence data that support the findings of this study have been deposited in the NCBI Sequence Read Archive (SRA) with the primary accession code SRP087753. The authors declare that all other data supporting the findings of this study are available within the article and its Supplementary Information files.

## Additional information

**How to cite this article:** Jia, X. *et al*. The role of alternative polyadenylation in the antiviral innate immune response. *Nat. Commun.* **8,** 14605 doi: 10.1038/ncomms14605 (2017).

**Publisher's note:** Springer Nature remains neutral with regard to jurisdictional claims in published maps and institutional affiliations.

## Supplementary Material

Supplementary Information Supplementary Figures, Supplementary Tables, Supplementary Methods and Supplementary References

## Figures and Tables

**Table 1:** GO analyses of APA genes between consecutive time points after VSV infection.

	**GO terms**	***P*****-value**
0–2 h	Enzyme-linked receptor protein signalling pathway	8.64 × 10^−5^
	Protein kinase cascade	3.13 × 10^−3^
	Response to endogenous stimulus	7.32 × 10^−3^
	Activation of MAPK activity	0.0149
	Regulation of JUN kinase activity	0.0184
	Positive regulation of signal transduction	0.0253
2–4 h	Phosphorylation	2.49 × 10^−4^
	Regulation of transcription	3.38 × 10^−4^
	Positive regulation of signal transduction	3.40 × 10^−4^
	JAK-STAT cascade	5.57 × 10^−3^
	Response to dsRNA	0.0198
	Endocytosis	0.025
4–8 h	Negative regulation of apoptosis	1.64 × 10^−4^
	Protein localization	7.46 × 10^−4^
	Inflammatory response	3.50 × 10^−3^
	Defence response	5.27 × 10^−3^
	Cell surface receptor-linked signal transduction	6.35 × 10^−3^
	Response to virus	0.034
	Endosome transport	0.037
	Immune response	0.0555
8–16 h	Protein transport	3.60 × 10^−5^
	Protein localization	5.42 × 10^−5^
	Regulation of cell death	3.52 × 10^−4^
	Immune response	4.32 × 10^−4^
	Inflammatory response	1.28 × 10^−3^
	Endocytosis	5.18 × 10^−3^
	Response to virus	5.25 × 10^−3^
	Positive regulation of T-cell proliferation	0.0116
16–24 h	Protein transport	4.85 × 10^−7^
	Establishment of protein localization	6.44 × 10^−7^
	Regulation of phosphate metabolic process	4.94 × 10^−6^
	Regulation of phosphorylation	1.31 × 10^−5^
	Regulation of kinase activity	2.13 × 10^−5^
	Cell death	5.47 × 10^−5^
	Apoptosis	9.92 × 10^−5^
	Endocytosis	6.50 × 10^−3^

APA, alternative polyadenylation; GO, gene ontology; VSV, vesicular stomatitis virus.

For testing GO item enrichment, a Fisher's exact test was performed, and the resulting *P*-values were corrected in the Benjamini–Hochberg sense.

**Table 2:** GO analyses of DEGs upon viral infection.

**GO terms**	**Human**		**Mouse**	
	**Count**	***P*****-value**	**Count**	***P*****-value**
Defence response	218	4.37 × 10^−5^	80	0.024768
Inflammatory response	129	1.64 × 10^−3^	54	7.086 × 10^−3^
Immune response	236	5.732 × 10^−3^	112	1.74 × 10^−5^
Response to virus	57	0.01064	18	0.055912
Response to wounding	182	0.024317	83	6.34 × 10^−4^
Cytokine–cytokine receptor interaction	90	0.031701	48	0.038692
Regulation of apoptosis	84	6.26 × 10^−6^	126	0.015092
Endocytosis	17	2.72 × 10^−4^	52	0.013111
Regulation of protein kinase cascade	17	6.642 × 10^−3^	52	8.638 × 10^−3^
Response to bacterium	68	8.566 × 10^−3^	30	0.076764

DEGs, differentially expressed genes; GO, gene ontology.

For testing GO item enrichment, a Fisher's exact test was performed, and the resulting *P*-values were corrected in the Benjamini–Hochberg sense.
