# Supplementary material for: The role of alternative polyadenylation in the antiviral innate immune response
Source: Nat Commun. 2017 Feb 24;8:14605. doi: 10.1038/ncomms14605 (PMC5333124; doi:10.1038/ncomms14605)
Supplement: Supplementary Information — Supplementary Figures, Supplementary Tables, Supplementary Methods and Supplementary References [file ncomms14605-s1.pdf]

Supplementary Methods

Linear trend (M^2) test to evaluate association between tandem 3' UTR length

To test multiple tandem poly(A) sites in one gene simultaneously and account for different polyadenylation effects with various tandem 3' UTR length, we adopted a method to test linear trend alternative to independence for two-way tables with ordered classifications similarly described as previously[1,2]. To test a gene with totally k tandem poly(A) sites across l time points, we performed the test as follows: 1) calculate tandem 3' UTR length for each of the k tandem poly(A) site; 2) put the number of reads for each tandem poly(A) site at each time point to an $l \times k$ contingency table: take tandem poly(A) sites as columns (from the site with shortest 3' UTR to that with longest); take the l by time series as rows; 3) if the total number of reads in the $l \times k$ table was less than 30, this gene was neglected for the test; otherwise 4) let the lengths of tandem 3' UTRs denote the scores for the columns; let the row NO. denote the row score, i.e. 1 for the 1st time point to be tested, 2 for the 2nd $\ldots l$ for the l th; 5) calculate Pearson correlation r (named as tandem 3' UTR isoform switch index, **TSI**, same as CTLI in Fu *et al*) by taking the number of reads in the table as the values and the scores for rows and columns as coordinates; 6) calculate a statistic: $M^2 = (n-1)r^2$; For large samples, this statistic is approximately chi-squared with df = 1 and *P* value can be obtained. When a gene with only two sites across two time points is considered, this test is reduced to be a canonical 2×2 chi-square test. A large *P* value suggests no association; a small *P* value with positive TSI implies a lengthening 3' UTR across the tested time points while a small *P* value with negative TSI implies a shortening 3' UTR.

Definitions of isoform-weighted 3' UTR length and normalized 3' UTR length (NUL)

For each gene at each time point with a tandem poly(A) site defined above, isoform-weighted 3' UTR length is defined as mean 3' UTR length weighted by read counts from each tandem 3' UTR isoform; and normalized 3' UTR length is defined as percentage of the isoform-weighted 3' UTR length relative to the longest tandem 3' UTR length across all time points. So through this normalization, two 3' UTR-fully-transcribed genes with different absolute 3' UTR lengths (for example, 500 nt vs. 5000 nt) were weighted equally (100 vs. 100 after normalization)

Flow cytometry analysis

Antibodies (CD14-FITC, CD11b-APC and F4/80-FITC) were purchased from Miltenyi Biotec. Freshly prepared cells were washed once in cold PBS and resuspended in cold staining buffer (PBS with 1% fetal bovine serum). Fluorescently labeled antibodies were added and cells were stained for 10 min at 4℃. Afterwards, cells were washed and resuspended in suitable amount of buffer. For each condition 10,000 cells were counted and analysis was performed on a flow cytometer (FACSCalibur).

RNA isolation, qRT-PCR and ELISA

Cells were harvested, washed once in cold PBS, and total cellular RNA was extracted using TRIzol reagent (Invitrogen) according to the manufacturer's protocol. For each sample, 1ug of total RNA was used in reverse transcription reactions using oligo-dT primers, random primers and SuperScript III (Invitrogen). One tenth of the cDNA was subjected to a 10ul quantitative RT-PCR performed in the Light Cycler480 instrument (using the 384-well module) with GoTaq qPCR Master Mix (Promega) according to the manufacturer's instruction. For APA analysis, primers were divided into 'common' and 'extended' 3' UTR regions (common primers detected both isoforms generated by proximal and distal sites usage, whereas extended primers detected only the isoform generated by cleavaging at the distal poly(A) site). The ratio between the extended and common primers of a transcript was indicative of the relative usage of the distal poly (A) sites. Expression quantities were normalized to beta-actin transcripts and the $2^{-\triangle\triangle Ct}$ method was used to calculate the relative levels of expression. For relative expression (fold), control samples were centred at 1. For ELISA assay, the concentrations of IFN-β and RANTES in culture supernatants were measured using the kits from R&D Systems according to the manufacturer's instructions.

Polysome profiling

Cells were treated for 5 min with cycloheximide at a final concentration of 100 μg/ml and then lysed with hypotonic buffer. Cytosolic extracts were ultra-centrifuged with a 5% to 50% sucrose gradient prepared using the Gradient Station, Biocomp Instruments. Fractions were collected with Gradient Station. Total RNA for each fraction was extracted from the cells using the QIAGEN

RNeasy® Mini kit, and maintained in an equal volume of RNase-free water. We then separated them into polysome-bound and unbound fractions, based on the OD254 values.

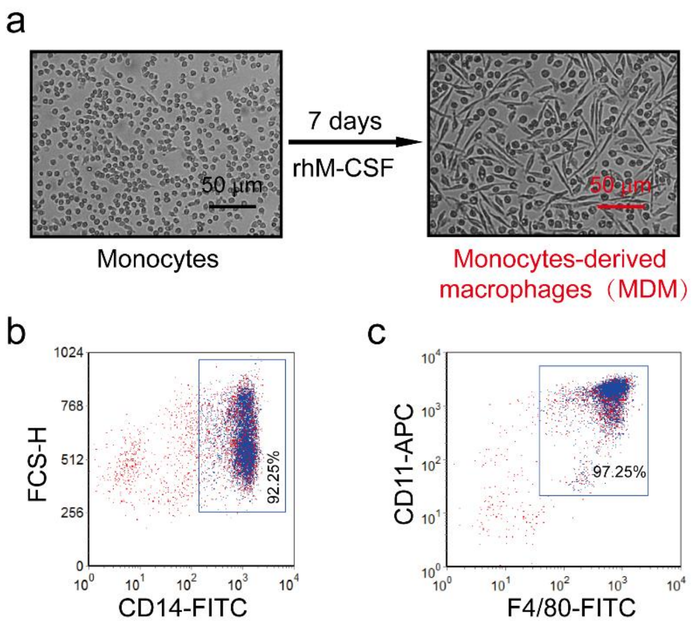

Supplementary Figure 1. Qualified cells were obtained. (**a**) CD14 positive cells were isolated from PBMC using MACS CD14 microbeads (left) and induced to macrophages using 50ng/ml rhM-CSF for7days(right). (**b**) CD14 monocytes were checked by flow cytometry and mouse peritoneal macrophages purity also identified by FCM (**c**).

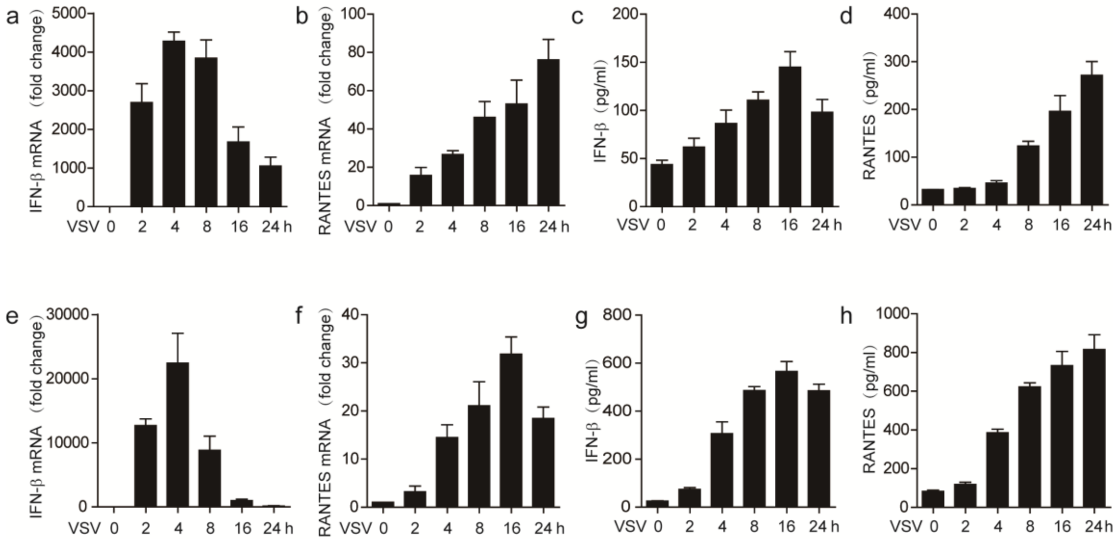

Supplementary Figure 2. Antiviral immunity was efficiently activated by VSV. MDMs (**a-d**) and mouse peritoneal macrophages (**e-h**) were infected with VSV at MOI: 0.5 for the indicated time points. Total RNAs or supernatants from the treated cells were harvested at different time points and measured by reverse transcription-quantitative PCR (qRT-PCR) or enzyme-linked immunosorbent assay (ELISA) to determine the expression of IFN-β and RANTES at mRNA level and protein level respectively. Error bars for qRT-PCR and ELISA were standard deviation based on two technical replicates.

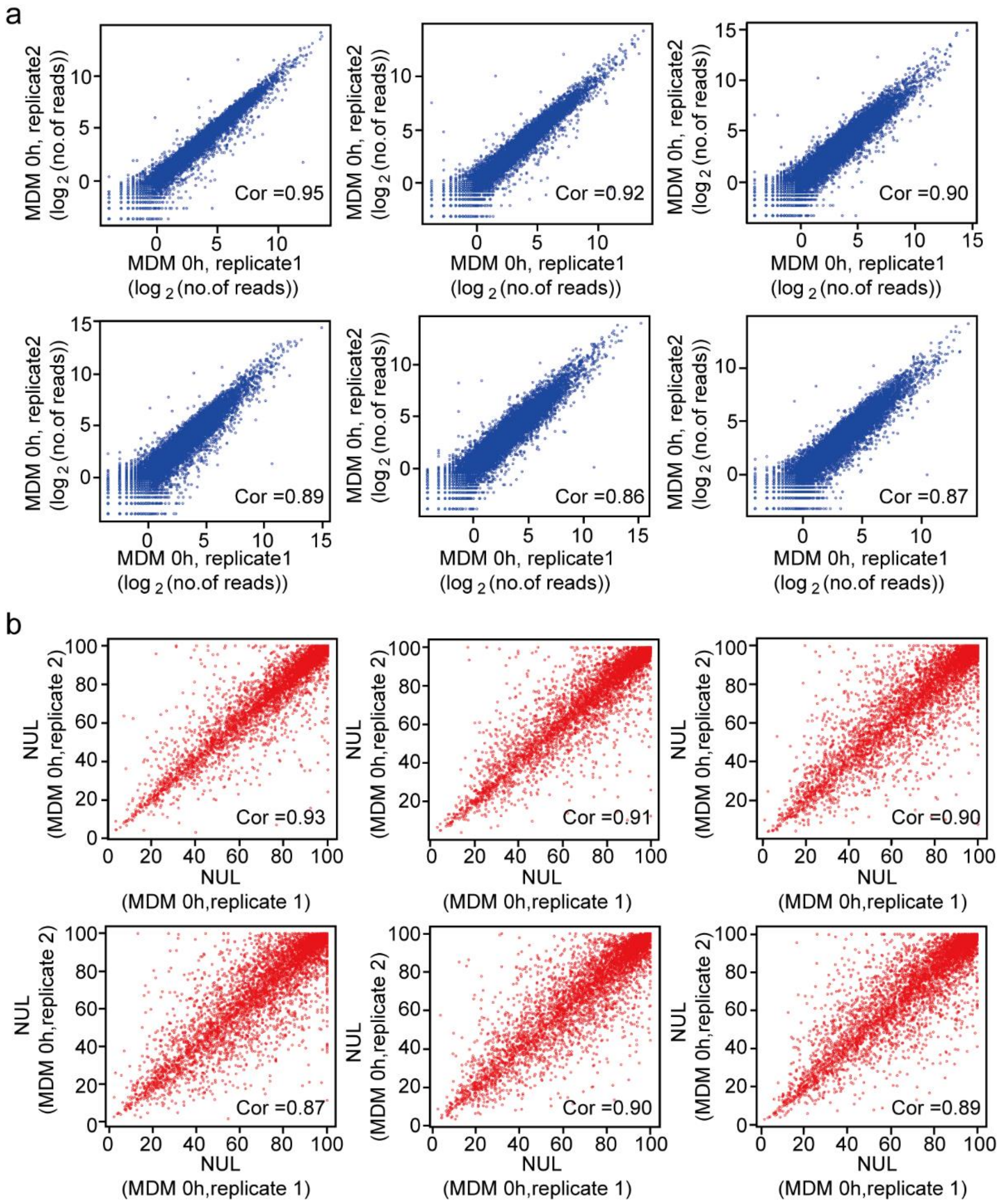

Supplementary Figure 3. Correlation of gene expression and normalized 3' UTR length (NUL) from two biological replicates of human MDMs. The number of reads that mapped to terminal exons of transcripts assigned to a particular gene was used as an estimate of the expression level of a gene.

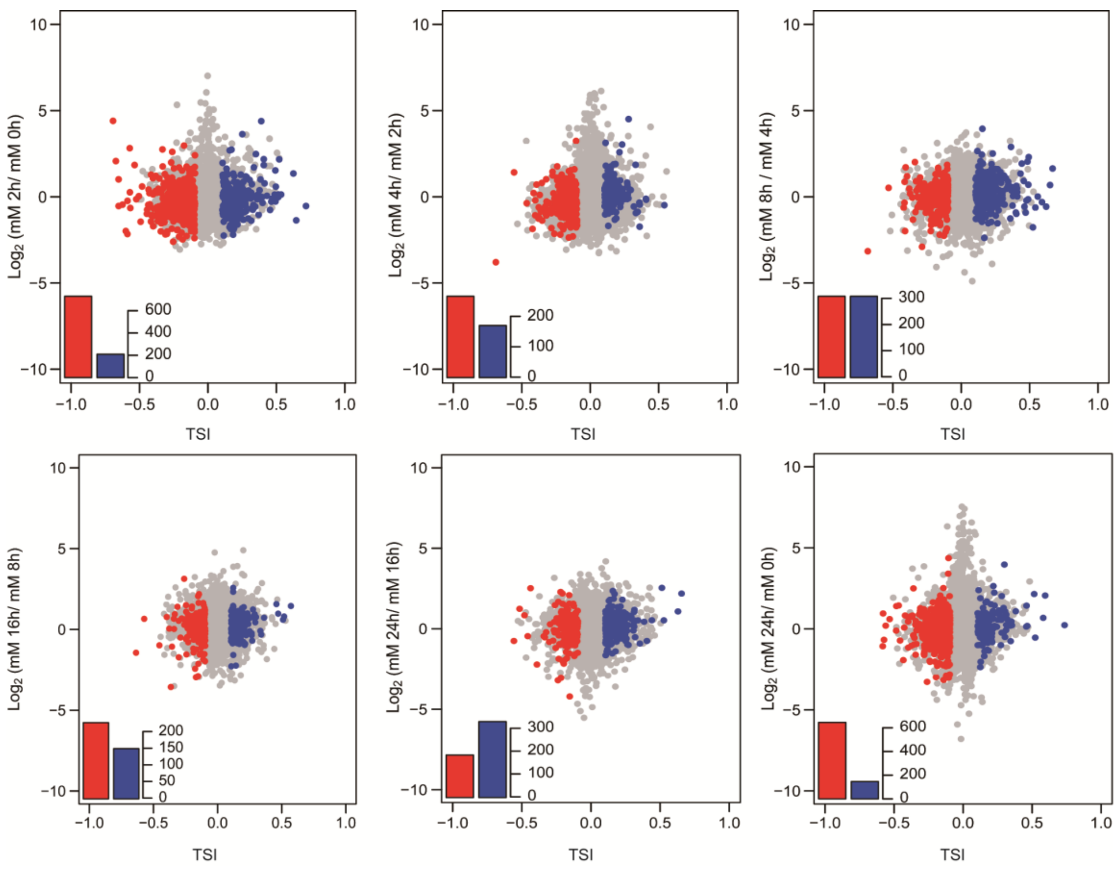

Supplementary Figure 4. Poly(A) sites switching of consecutive time points in murine species. The x-axis denotes TSI, a larger positive value indicates that longer tandem 3' UTRs are prone to be used in latter time point samples. Genes with significant switching to longer (blue) or shorter (red) tandem 3' UTRs in latter time point samples (FDR=0.01) are colored. The y-axis denotes the logarithm of the expression level of genes from latter time point sample relative to former time point sample.

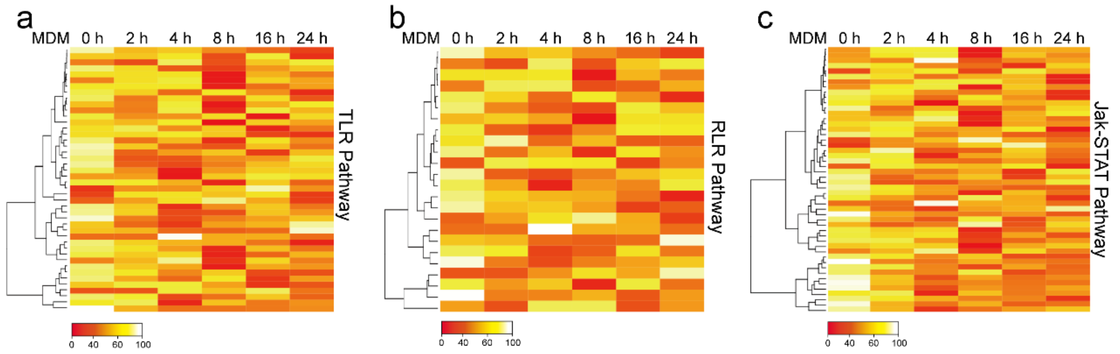

Supplementary Figure 5. Heat map of normalized weighted 3' UTR length of genes involved in indicated pathways. Each row corresponds to a gene, and each column corresponds to a time point sample. White indicates longer 3' UTR isoform and red the shorter 3' UTR isoform.

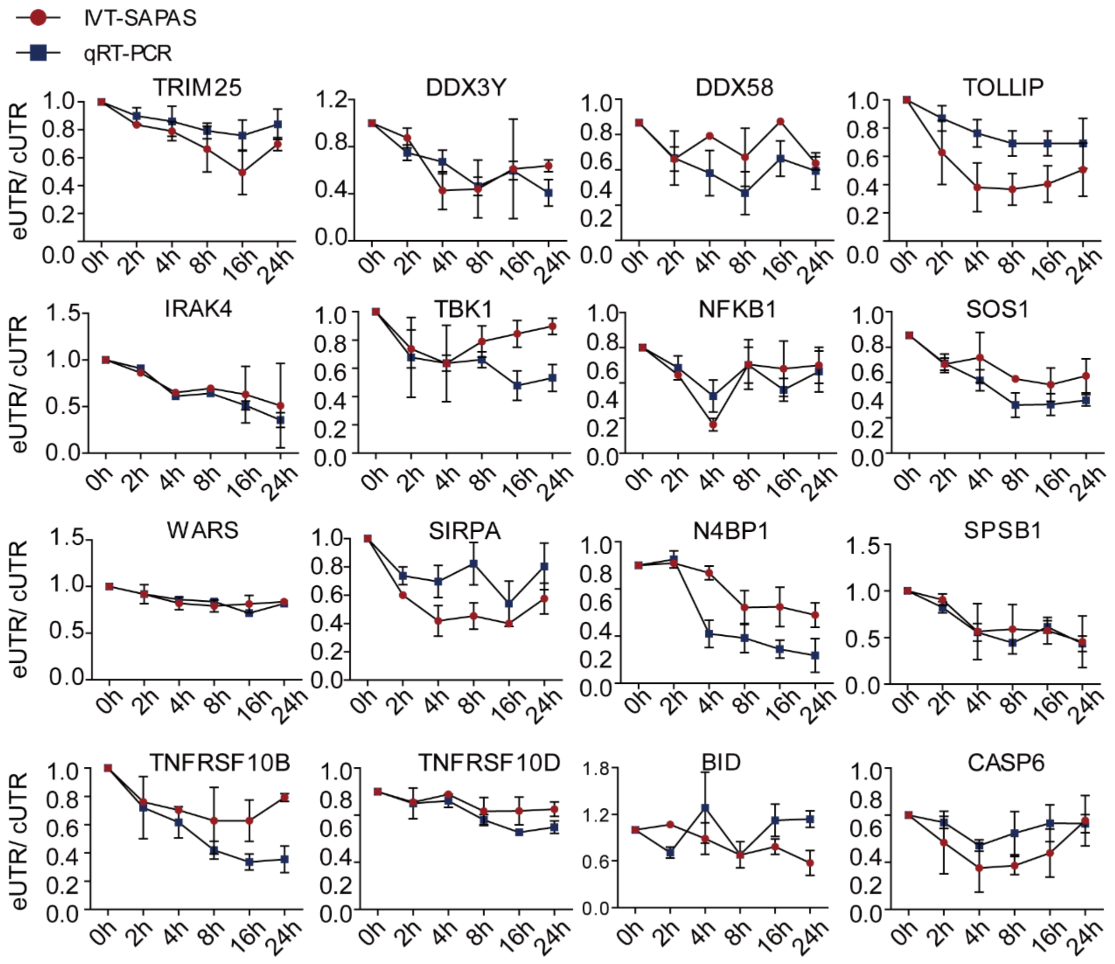

Supplementary Figure 6. qRT-PCR validation of APA genes involved in antiviral signaling network. VSV infected MDMs at an MOI of 0.5 at six time points and qRT-PCRs were performed. The x-axis denotes six infection time points and the y-axis denotes e UTR/ c UTR, which represents the ratio of longer 3' UTR isoforms to total mRNA isoforms. Sequencing data are presented as mean ± SD from two biological replicates of IVT-SAPAS. qRT-PCR data are presented as mean ± SD of triplicate determinations from three independent experiments.

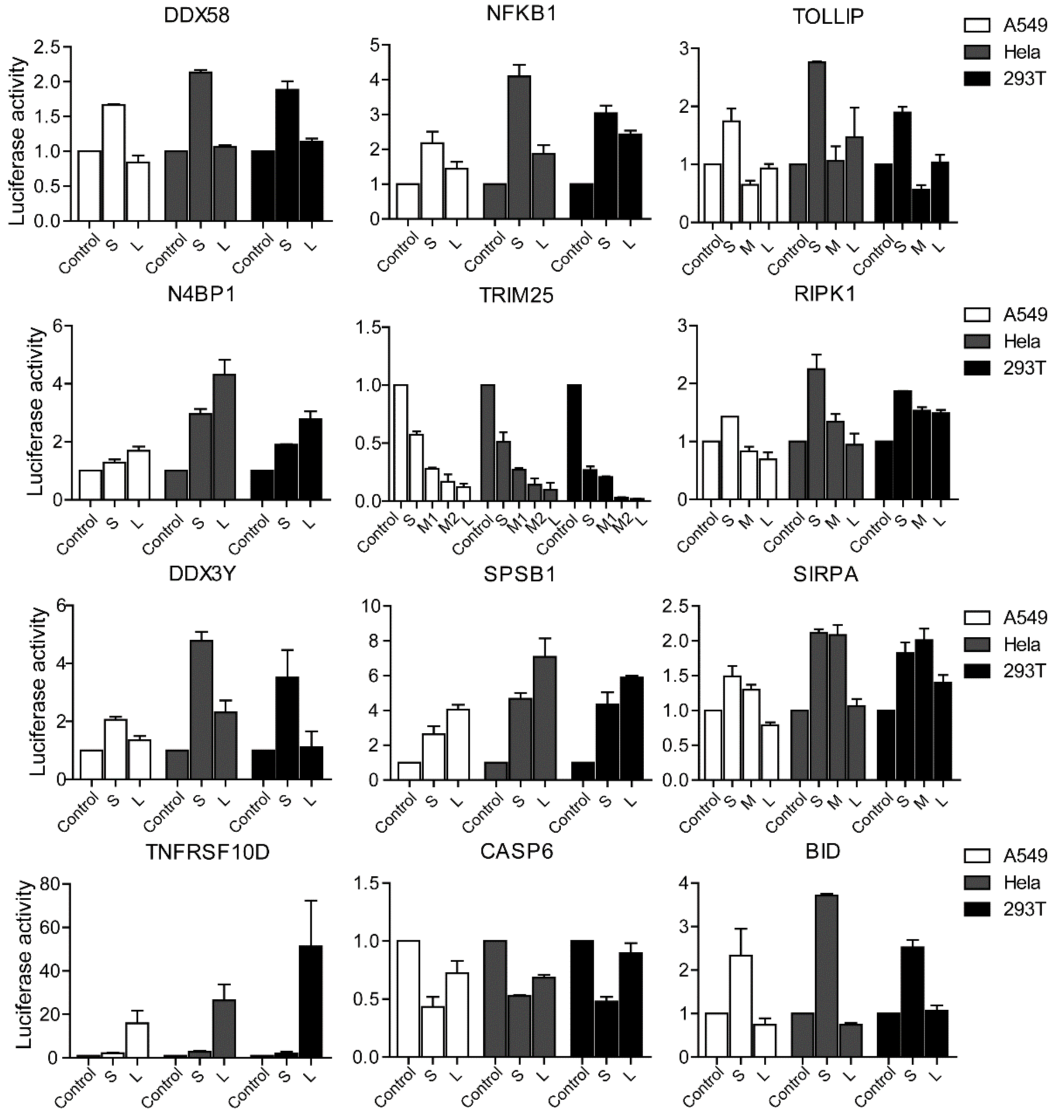

Supplementary Figure 7. Luciferase assays conducted in three distinct cell lines showed coherent expressions of different 3'UTR mRNA isoforms. psiCHECK-2 or psiCHECK-3' UTR constructs were transfected into A549 cells, Hela cells, and 293T cells. The luciferase expression of a reporter possessing the 3' UTRs of target genes was normalized to that of psiCHECK-2. Histograms show the mean reporter activity values, with error bars indicating standard deviation (n = 9, performed as three independent experiments with three transfections of each reporter).

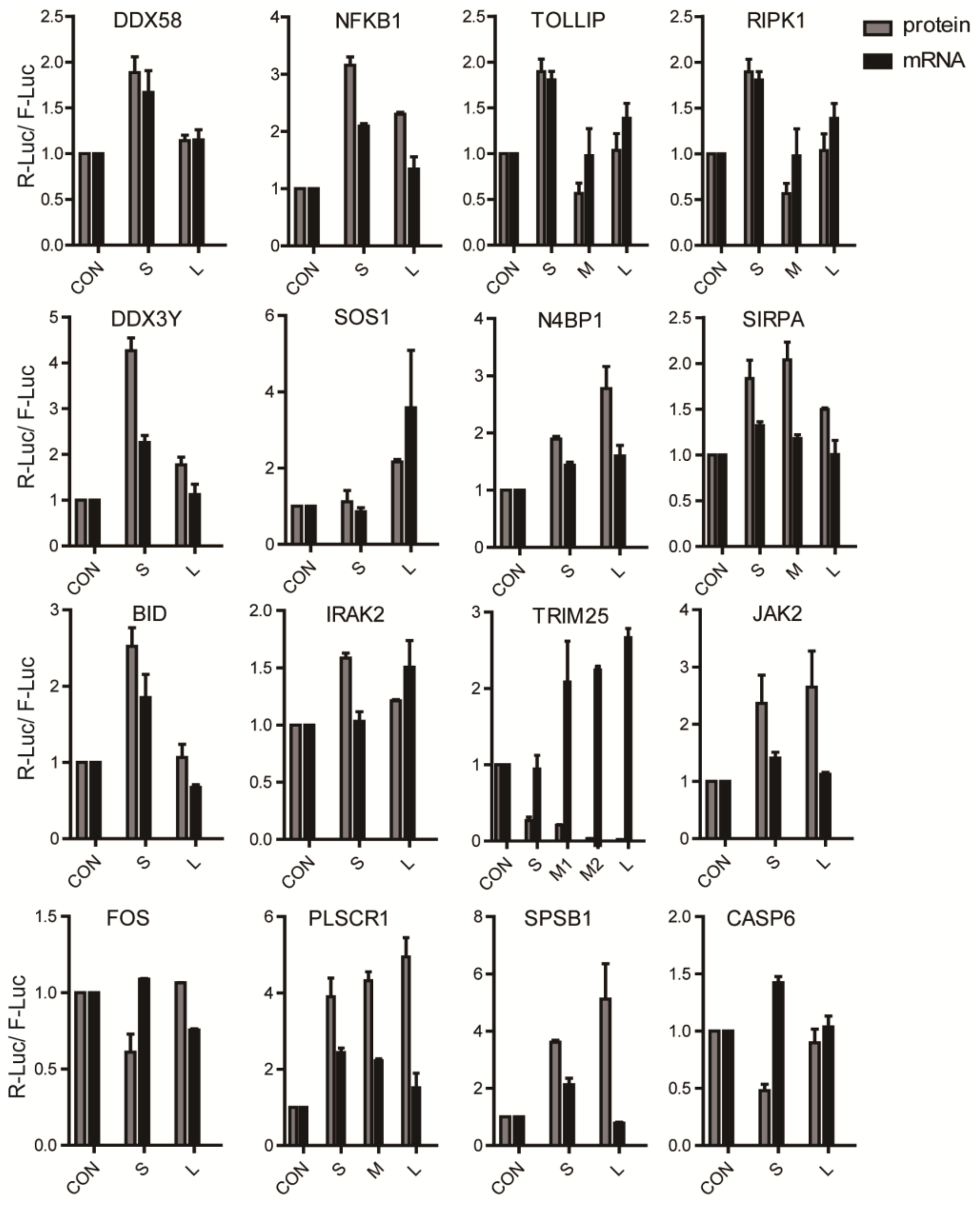

Supplementary Figure 8. The effect of tandem 3' UTR on protein production and mRNA abundance. psiCHECK-2 or psiCHECK-3' UTR constructs were transfected into 293T cells, and half of the cells were lysed for reporter gene assays, the remaining cells were collected for qRT-PCR analysis 24 hours later. Luciferase expression of a reporter possessing 3' UTRs of target genes is normalized to that of psiCHECK-2. Data are presented as mean ± SD of triplicate determinations from three independent experiments.

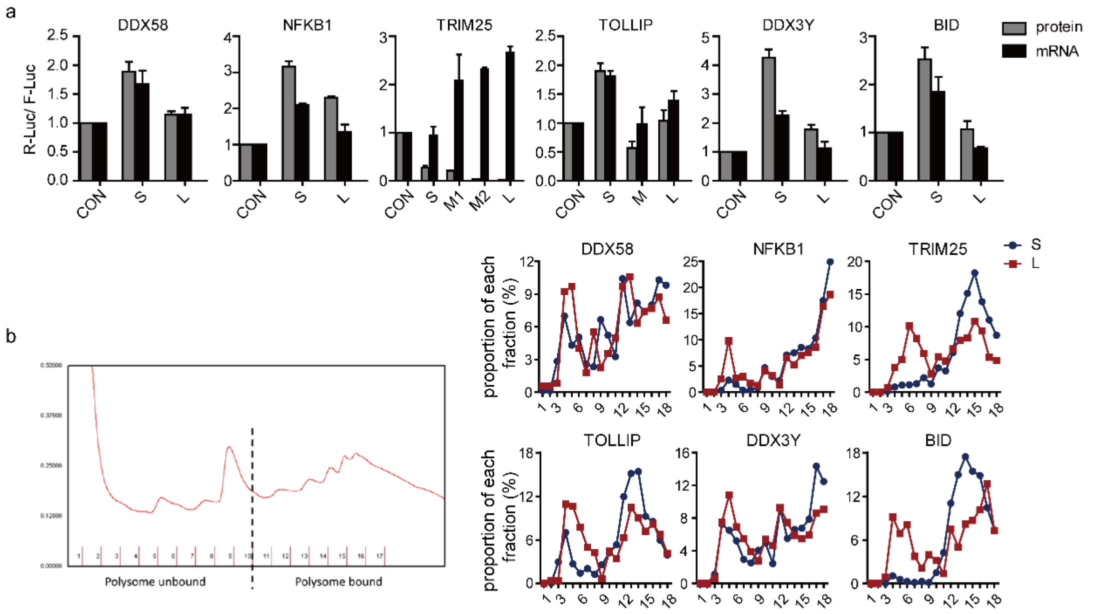

Supplementary Figure 9. Consequence of tandem 3’ UTR on protein production, mRNA abundance, and translation efficiency. (**a**) The effect of tandem 3’ UTR on protein production and mRNA abundance of six genes, same as Supplementary Fig. 8. (**b**) Polysome profiling assay were performed to estimate the translation efficiency of the endogenous mRNAs in 293T cells for six pivotal genes. The vertical dashed line shows the boundary of the polysome-bound and unbound fractions which indicates high translation efficiency and low translation efficiency, respectively. Two pairs of primers were designed for the common (S) and extended (L) 3’ UTRs of the specific gene and used to measure the level of total mRNA and the isoforms with longer 3’ UTR. The proportion of mRNA of each fraction measured by qRT-PCR and the specified mRNA level in each fraction was calculated as a percentage of the total.

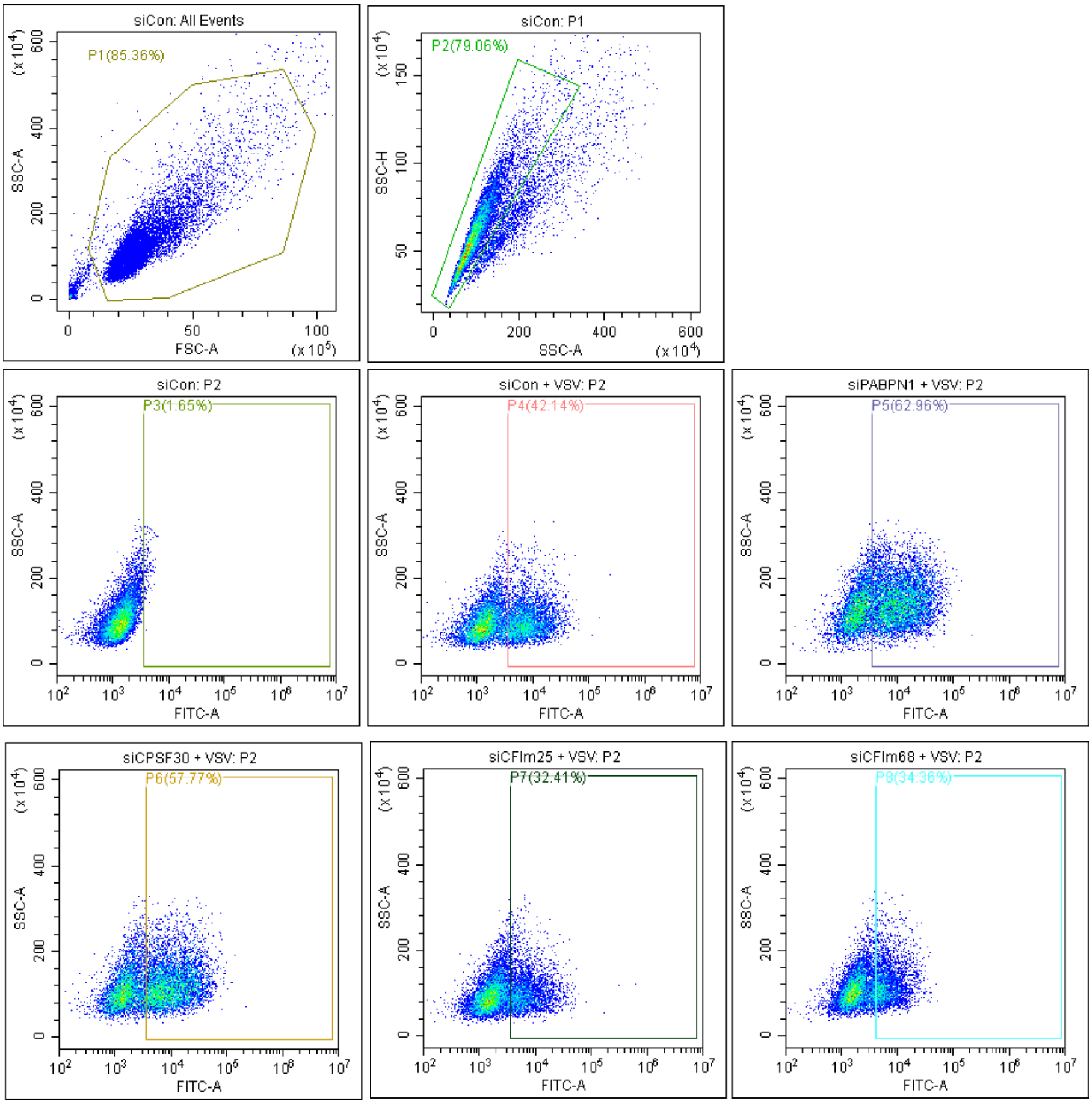

Supplementary Figure 10. Representative flow cytometric dot plots to account for FACS gating strategies. Representative flow cytometric dot plots from one of three independent experiments.

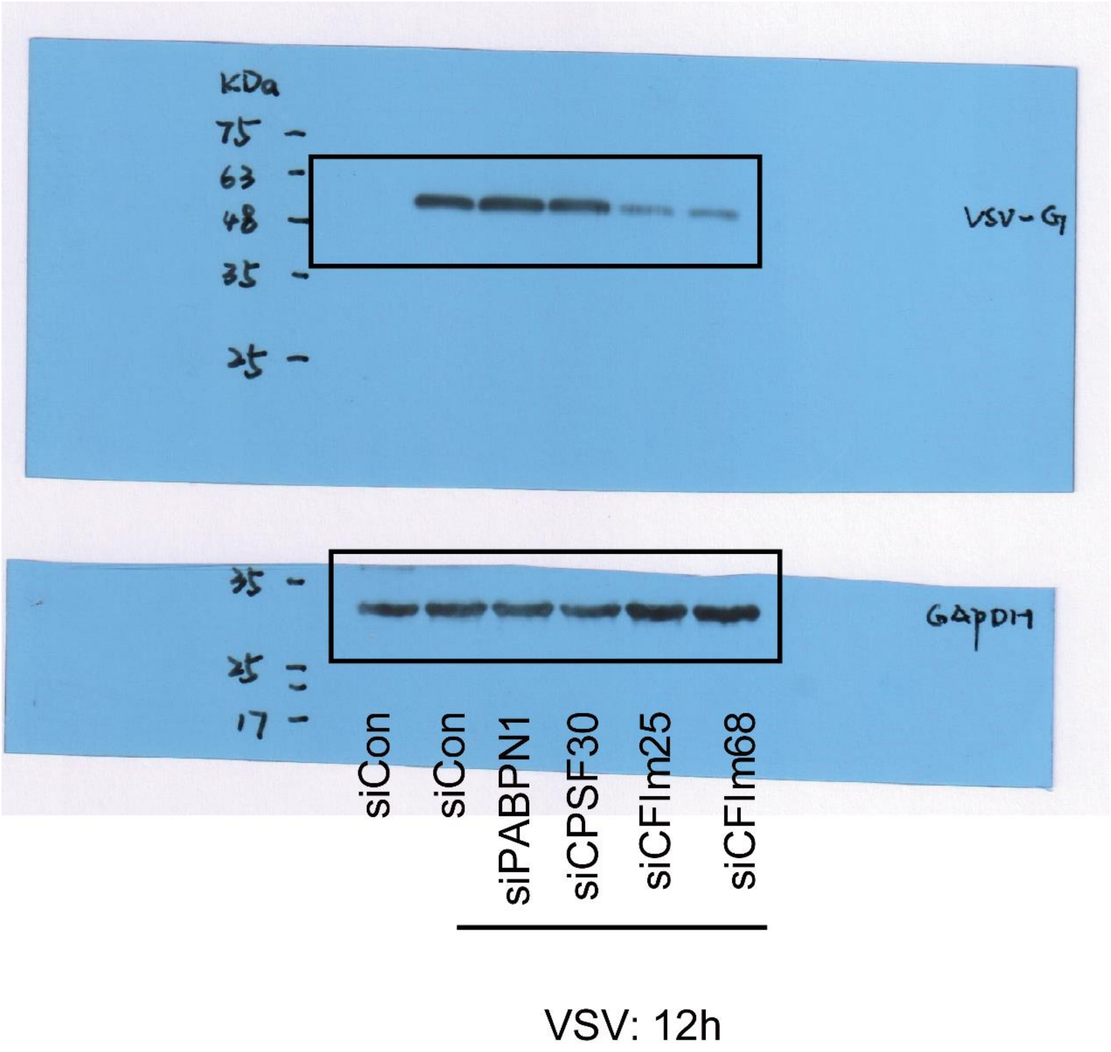

Supplementary Figure 11. Full scan blots of Fig.6 d.

The cropped areas used in Fig. 6d are marked by black boxes. One representative data of three independent experiments with similar results are shown.

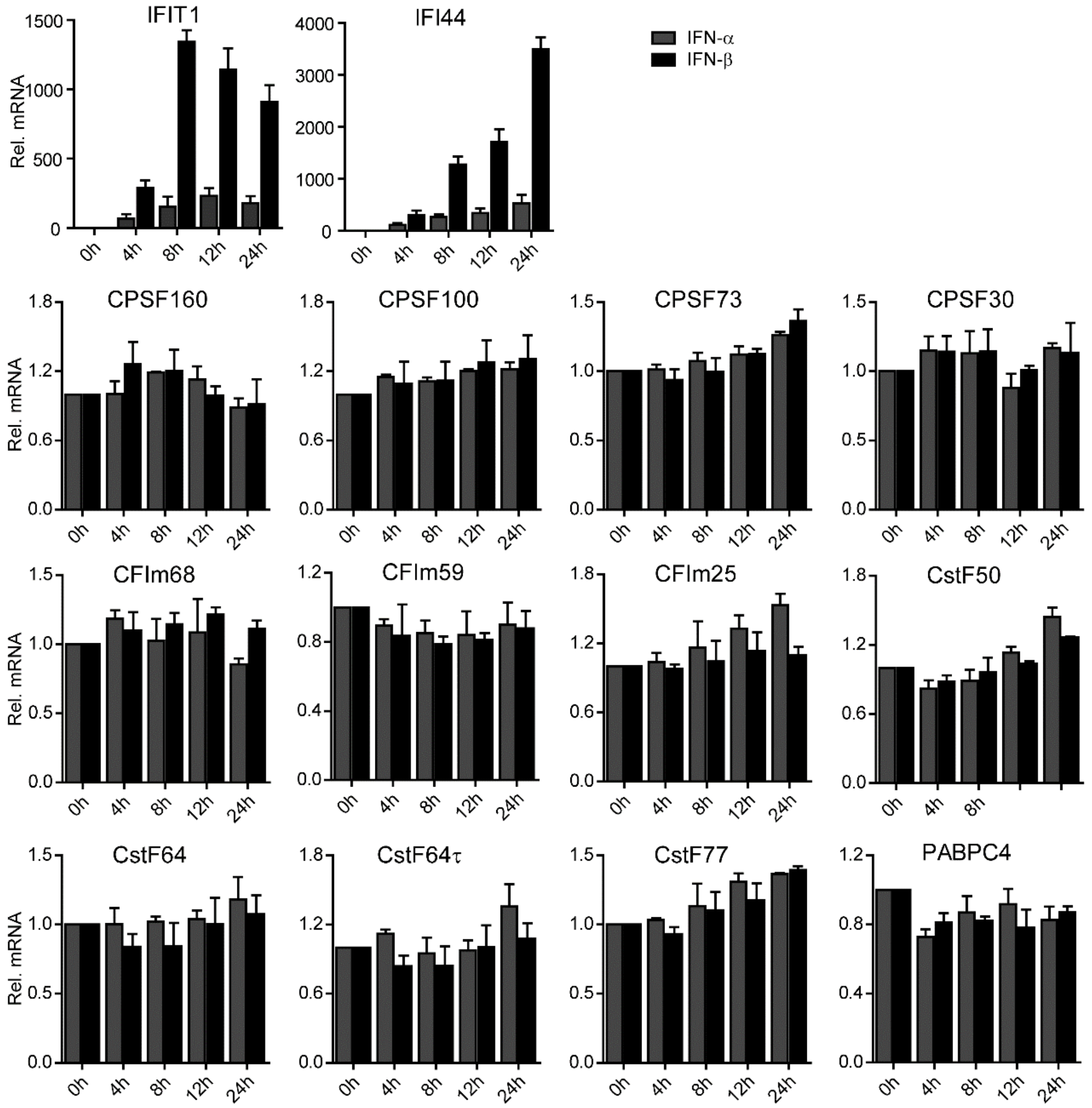

Supplementary Figure 12. mRNA changes of core 3' processing factors in IFNα or IFNβ stimulated THP1 cells. Data were obtained by qRT-PCR. *IFIT1* and *IFI44* served as positive controls. The x-axis denotes five time points post infection and the y-axis denotes the relative expression to 0h. Data are presented as mean ± SD of triplicate determinations from three independent experiments.

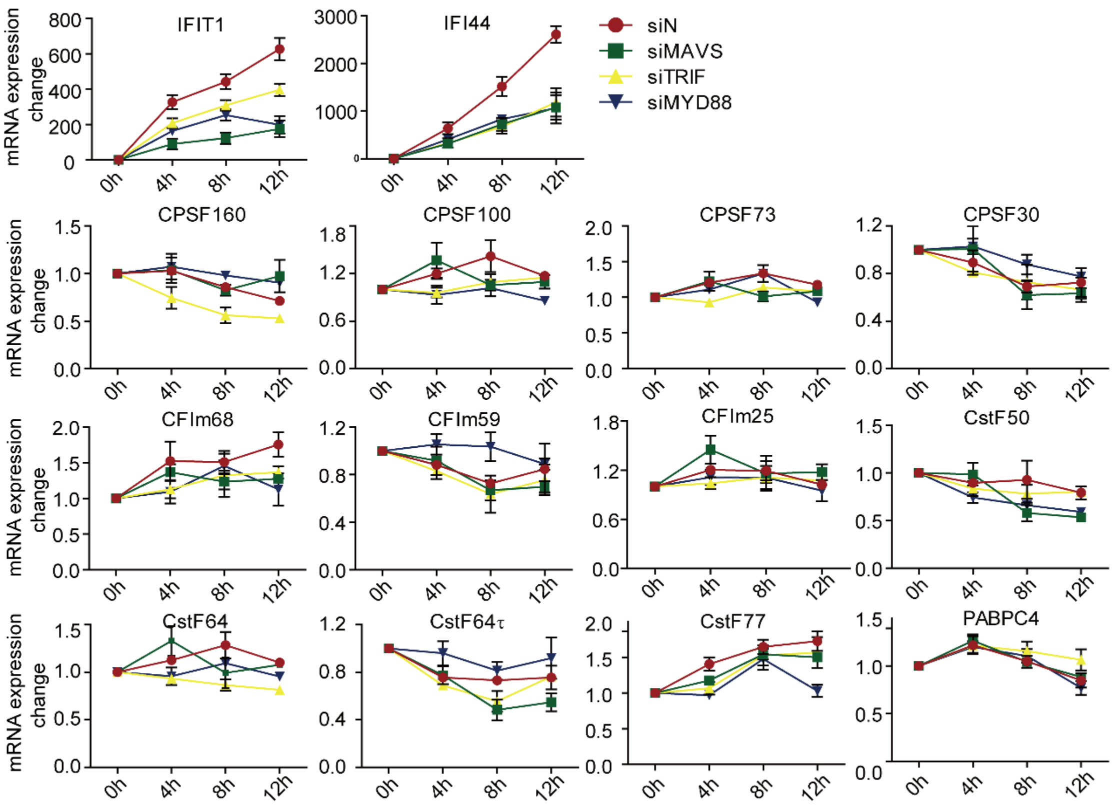

Supplementary Figure 13. mRNA expression changes of core 3' processing factors in the depletion of MyD88, TRIF and MAVS. A549 cells were transfected with indicated siRNAs for 48 h, then the cells were infected with VSV at an MOI of 1 for four time points. qRT-PCR assays were conducted, *IFIT1* and *IFI44* served as positive controls. The x-axis denotes four time points post VSV infection and the y-axis denotes the relative expression to 0h. Data are presented as mean ± SD of three independent experiments.

Supplementary Table 1. Summary statistics for homo species IVT-SAPAS sequencing and mapping to genome (h19).

	0h	2h	4h	8h	16h	24h	combined
raw reads	4,8058,713	45,985,639	42,156,899	37,963,131	44,222,663	39,570,443	257,957,488
after polyNT control	45,281,427	43,654,168	40,538,630	34,656,388	42,092,514	37,317981	243,541,108
Mapped to genome	41,328,861	39,542,461	36,033,540	30,343,272	36,676,305	32,972,415	216,896,854
Uniquely mapped to genome	29,989,060	27,765,190	26,380,585	22,303,026	27,517,531	26,139,929	160,095,321
Mapped to nuclear genome	23,179,045	19,992,621	19,136,791	17,145,702	22,326,713	22,917,523	124,698,395
After IP filter	16,118,542	13,749,336	15,986,032	10,587,795	17,605,811	16,233,965	90,281,481
Genes sampled by reads	16,081	16,232	16,014	16,593	16,583	16,380	18,888
Cleavage clusters	392,491	311,446	164,209	499,280	285,593	469,647	1,185,138
Known polyA sites sampled	25,744	25,103	24,577	23,179	25,322	25,665	31,213
Putative novel polyA sites	366,747	286,343	139,632	476,101	260,271	443,982	1,153,925
Genes sampled by cleavage clusters	14,366	14,322	14,341	14,657	14,394	14,811	17,122

Supplementary Table 2. Summary statistics for murine species IVT-SAPAS sequencing and mapping to genome (mm9).

	0h	2h	4h	8h	16h	24h	combined
raw reads	49,718,224	46,787,819	49,423,822	44,335,326	50,050,234	42,220,848	564,259,676
after polyNT control	49,584,759	46,661,738	49,280,868	44,145,154	49,936,347	42,114,537	535,840,080
Mapped to genome	45,719,132	41,000,352	42,393,397	40,521,312	45,846,930	38,635,554	471,589,367
Uniquely mapped to genome	38,426,287	38,426,287	34,302,184	34,357,094	39,149,016	32,811,822	422,756,162
Mapped to nuclear genome	37,190,437	31,125,539	32,932,566	33,659,732	38,486,276	31,888,922	386,564,353
After IP filter	33,599,772	25,493,792	27,733,979	30,243,968	35,125,993	29,083,377	181,398,638
Genes sampled by reads	19,585	21,961	22,073	20,296	17,325	16,517	23,409
Cleavage clusters	337,276	1,057,299	1,005,642	407,490	214,136	259,036	2,389,134
Known polyA sites sampled	20,971	20,765	20,453	20,397	21,017	20,846	25,643
Putative novel polyA sites	316,305	1,036,534	985,189	387,093	193,119	238,190	2,363,491
Genes sampled by cleavage clusters	16,951	19,742	19,825	17,352	14,896	14,500	21,960

Supplementary Table 3. mRNA abundance level of IFNB1.

Gene symbol	PAS	0h	2h	4h	8h	16h	24h
hIFNB1	1	1.86	336.235	524.276	303.742	257.759	22.735
mIFNB1	1	0.328	45.845	131.658	102.608	3.316	0.855

Supplementary Table 4. mRNA abundance level of selected genes.

Gene symbol	PAS	MDM_0h	MDM_2h	MDM_4h	MDM_8h	MDM_16h	MDM_24h
TICAM1	1	83.754	252.158	367.824	150.265	334.549	267.034
STAT1	1	38.278	28.438	167.212	351.149	184.035	441.979
IRF7	1	10.298	32.803	35.907	71.024	94.402	66.158
IKBKB	1	0.062	0.363	0	0.189	0.057	0.37
MAVS	1	2.17	0.509	1.378	0.471	0.853	1.664
TYK2	1	133.138	184.518	193.171	65.828	129.22	122.4
PIAS4	1	0.248	0.582	0.939	0.661	0.966	1.295
IL15RA	1	1.922	3.708	5.82	13.224	17.551	43.43
IFNGR1	1	244.189	54.768	34.908	26.539	41.294	202.91

Supplementary References

1. Fu, Y. *et al.* Differential genome-wide profiling of tandem 3' UTRs among human breast cancer and normal cells by high-throughput sequencing. *Genome Res.* **21,** 741-747 (2011).
2. Li, Y. et al. Dynamic landscape of tandem 3' UTRs during zebrafish development. *Genome Res.* **22,** 1899-1906 (2012).
